# Une infection par le SARS-CoV-2 chez un patient sous hydroxychloroquine et anti TNF α pour rhumatisme inflammatoire chronique

**DOI:** 10.11604/pamj.supp.2020.35.134.25011

**Published:** 2020-08-05

**Authors:** Saadia Ait Malek, Imane El Bouchti

**Affiliations:** 1 Service de Rhumatologie, Centre Hospitalier Universitaire Mohamed VI, Marrakech, Maroc

**Keywords:** Hydroxychloroquine, urticaire, SARS-CoV-2, rhumatisme inflammatoire chronique, Hydroxychloroquine, urticaria, SARS-CoV-2, chronic inflammatory rheumatic disease

## Abstract

L´hydroxychloroquine est un agent utilisé comme traitement mais aussi envisagé comme prophylaxie pour l'infection au SARS-CoV-2. Nous rapportons le cas d´un patient qui a développé COVID-19 pendant qu´il était sous hydroxychloroquine pour une connectivité mixte associée à une spondyloarthrite. Bien que d´autres travaux soient nécessaires pour pouvoir tirer des conclusions, cela amène à s´interroger sur le rôle protecteur de ce médicament contre l´infection. Sont-ils vraiment protégés contre le COVID-19 ou développeront des formes pauci-symptomatiques?

## Abstract (en)

Hydroxychloroquine is an agent used as a treatment but also considered as a prophylaxis for SARS-CoV-2 infection. We report the case of a patient who developed COVID-19 while on hydroxychloroquine for mixed connectivitis associated with spondyloarthritis. Although more work is needed before any conclusions can be drawn, this raises questions about the protective role of this drug against infection. Are they really protected against COVID-19 or will they develop pauci-symptomatic forms?

## Introduction

Les rhumatismes inflammatoires chroniques (RIC) nécessitent une attention particulière avec la pandémie COVID-19. Ils sont considérés à risque d'infections graves en raison d'une altération du système immunitaire, de l'utilisation des immunosuppresseurs et les comorbidités [1]. Cependant, Il n'est pas certain qu´ils courent un risque plus élevé de contracter le virus ou de présenter une forme sévère [2]. Jusqu´à présent, il n´existe pas de traitement antiviral spécifique pour le COVID-19. La progression des connaissances sur la pathogenèse de l'infection ouvre la voie à l'utilisation de certains médicaments utilisés en rhumatologie pour traiter également le COVID-19.

## Patient and Observation

**Figure 1:**
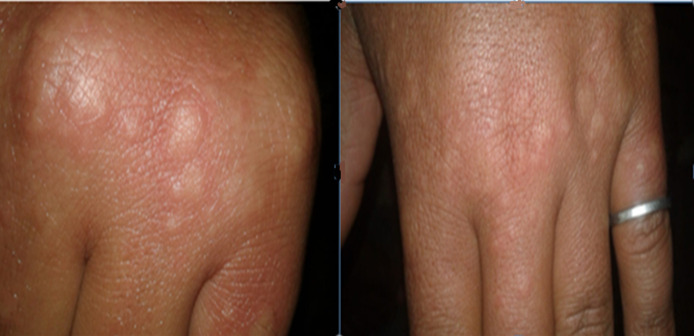
éruption cutanée faite de papules arrondies œdémateuses, roses, centre plus clair, et contours bien limités

Un homme de 20 ans suivi pour une spondyloarthrite axiale associée à une connectivité mixte, traité par hydroxychloroquine à 400 mg /j et Etanercept 50mg/semaine. Le patient présente 7 jours après le contact avec son père ayant COVID-19, une atteinte cutanée au niveau des mains évoquant une urticaire aigue (Figure 1) sans fièvre ni signes respiratoires ni digestifs. Le test PCR s'est révélé positif. À noter que le patient a eu son injection d´Etanercept 2 jours avant le diagnostic de l´infection COVID-19. A son admission à l'hôpital, le patient est apyrétique, sans détresse respiratoire ni hémodynamique. La tomodensitométrie thoracique n'a pas montré d´infiltrat. Le bilan biologique est normal. Au cours de l´hospitalisation, son hydroxychloroquine (HCQ) a été augmenté à 600 mg/j pendant 10 jours, en association à l´azithromycine 500mg au 1 er jour puis 250 mg/j pendant 6 jours. L´évolution était favorable, l´éruption cutanée a disparu en 5 jours. Deux PCR de contrôle étaient négatives. Le patient a été déclaré guéri.

## Discussion

Ce cas présente plusieurs particularités, d´abord la survenue de l´infection COVID sur un terrain immunodéprimé recevant de l´hydroxychloroquine et l´anti TNF-α. La révélation de l´infection par une atteinte cutanée isolée ainsi que l´évolution bénigne. Dans une étude internationale multicentrique, en cours, sur un total de 872 RIC atteints de covid: la répartition était la suivante: PR (39,11%), lupus (15,6%), RP (10,89%), SPA (7,11%), vascularite (6,77%), Sjogren (4%). Les traitements en cours étaient: csDMARDs (65,25%), b-DMARD (32,5%), antipaludéens (23,95%), inhibiteur du Janus kinase (5,39%) [3]. Le TNF est présent dans le sang des patients atteints de COVID-19 et ses niveaux sont même plus élevés chez ceux atteints d'une forme grave [4]. La littérature reste très limitée concernant les traitements possibles de la tempête cytokinique en matière de l´anti TNF-α. Dans des modèles animaux la neutralisation du TNF-α offre une protection contre l´infection au SARS-CoV2 [5]. À ce jour, un seul essai clinique randomisé évaluant l'adalimumab dans COVID-19 a été enregistré (ChiCTR2000030089). Plusieurs études évaluent la place de l´anti IL6 dans le traitement du COVID-19 [6]. La réflexion sur un rôle préventif de l´HCQ lorsqu´il est prescrit de façon chronique est une question actuellement non résolue, d´un intérêt particulier pour les rhumatologues. D'après de petites séries de cas publiés, trois des huit patients covid positif prenaient de l´HCQ, ce qui ne semblait pas empêcher l'infection dans ces cas [2].

## Conclusion

Les cliniciens doivent souligner que ces traitements ne confèrent pas une protection contre l´infection. Des essais cliniques randomisés s´avèrent nécessaires.
